# PARP inhibitor ABT-888 affects response of MDA-MB-231 cells to doxorubicin treatment, targeting Snail expression

**DOI:** 10.18632/oncotarget.3634

**Published:** 2015-04-13

**Authors:** Germano Mariano, Maria Rosaria Ricciardi, Daniela Trisciuoglio, Michele Zampieri, Fabio Ciccarone, Tiziana Guastafierro, Roberta Calabrese, Elisabetta Valentini, Agostino Tafuri, Donatella Del Bufalo, Paola Caiafa, Anna Reale

**Affiliations:** ^1^ Department of Cellular Biotechnologies and Haematology, “Sapienza” University of Rome, Italy; ^2^ Experimental Chemotherapy Laboratory, Regina Elena National Cancer Institute, Rome, Italy; ^3^ Pasteur Institute-Fondazione Cenci Bolognetti, Rome, Italy; ^4^ Department of Clinical and Molecular Medicine, “Sapienza” University of Rome, Italy

**Keywords:** PARP inhibitors, chemoresistance, Snail, breast cancer

## Abstract

To overcome cancer cells resistance to pharmacological therapy, the development of new therapeutic approaches becomes urgent. For this purpose, the use of poly(ADP-ribose) polymerase (PARP) inhibitors in combination with other cytotoxic agents could represent an efficacious strategy. Poly(ADP-ribosyl)ation (PARylation) is a post-translational modification that plays a well characterized role in the cellular decisions of life and death. Recent findings indicate that PARP-1 may control the expression of Snail, the master gene of epithelial-mesenchymal transition (EMT). Snail is highly represented in different resistant tumors, functioning as a factor regulating anti-apoptotic programmes. MDA-MB-231 is a Snail-expressing metastatic breast cancer cell line, which exhibits chemoresistance properties when treated with damaging agents. In this study, we show that the PARP inhibitor ABT-888 was capable to modulate the MDA-MB-231 cell response to doxorubicin, leading to an increase in the rate of apoptosis. Our further results indicate that PARP-1 controlled Snail expression at transcriptional level in cells exposed to doxorubicin. Given the increasing interest in the employment of PARP inhibitors as chemotherapeutic adjuvants, our *in vitro* results suggest that one of the mechanisms through which PARP inhibition can chemosensitize cancer cells *in vivo*, is targeting Snail expression thus promoting apoptosis.

## INTRODUCTION

Breast cancer is the most common malignant tumor in women. Despite advances in diagnosis and treatment approaches, the mortality due to breast cancer still remains very high. This is attributable to the fact that cancer cells are able to develop mechanisms of resistance to the therapeutic treatment, a process known as chemoresistance [1, 2]. In particular, the treatment of some kind of triple negative breast cancer (TNBC) -so called because the cancer cells lack receptor for estrogen (ER^−^) and progesterone (PR^−^) and do not express the human epidermal growth factor receptor 2 (HER2^−^)- is quite complex because to an initial response follows a resistance to therapy [3].

Epithelial-mesenchymal transition (EMT) is a transdifferentiation program that is required for tissue morphogenesis during embryonic development and abnormally activated during tumor progression [4–6]. Through EMT, epithelial cells lose their cell-cell adhesion molecules, their polarity, rearrange their cytoskeleton and become prone to migrate [7]. Recent evidence now indicates that EMT of tumor cells not only promotes the development of metastases, but also contributes to drug resistance [8–12]. Ultimately, Snail, the fundamental member of the family of Snail transcriptional factors [13], has emerged as factor able to increase the resistance of cancer cells [14, 15]. It has been demonstrated that aberrant expression of Snail promotes resistance to programmed cell death in MCF-7 cells elicited by doxorubicin [14]. Moreover, Snail has been reported to be sufficient to promote mammary tumor recurrence *in vivo* and high levels of Snail predict decreased relapse-free survival in women with breast cancer [16]. Other studies have shown that Snail confers resistance to cell death induced by lack of survival factors and by pro-apoptotic signals [17] and that Snail downregulation increases cell death in colon tumors in a mouse model [18]. Snail exerts its function not only through the repression of epithelial genes such as *CDH1* (E-cadherin) [19] but also through repression of multiple factors with important functions in apoptosis such as *TP53*, *BID*, and *Caspase-6* [14, 20] or *PTEN*, a negative effector of the PI3K pathway [21].

Snail expression is regulated at transcriptional level by growth factors and various signaling molecules [22] as well as by Snail protein itself [23]. In addition, some post-translational modifications influence Snail stability, subcellular localization and activity [24–26]. Moreover, it has been shown that the expression of Snail can be directly activated by the enzyme PARP-1 at transcriptional level [27] and that the modification of Snail by PARP activity stabilizes Snail protein at post-translational level [24].

Recent findings have revealed that PARP-1 plays a role in EMT and metastasis formation by affecting the expression of epithelial and mesenchymal genes [28–31]. To investigate the relationship between PARP-1 and Snail, we have to keep in mind the pleiotropic actions of PARP-1. PARP-1 is an abundant and ubiquitous nuclear enzyme [32, 33], which is implicated in multiple pathways involved in the regulation of gene expression [34–39]. In response to stresses that are toxic to the genome, PARP-1 binds single or double-stranded DNA breaks and its activity increases with the aim to maintain genomic integrity [40, 41]. Massive PARP-1 activation, however, consumes NAD^+^ and ATP, leading to energy failure and cell death [42, 43]. A delicate PARP-1 activity equilibrium exists within cells where any deviation, either hyper- or hypo-activity can change the cell decision of life or death [44]. PARP-1 is an attractive anticancer target [45, 46] and PARP inhibitors used as monotherapy, induce cell death in tumors with non-functional homologous recombination because of defective BRCA pathways [47–49]. PARP inhibitors are also used in combination with chemotherapeutic agents to strengthen the effect of DNA damage in sporadic tumors [50–52]. As previously described [53–55], PARP inhibitors, when used in combination with doxorubicin (doxo), are able to chemosensitize p53-mutated, TNBC MDA-MB-231 cells. In this paper, we aim to explore whether this chemosensitizing effect may depend on the regulation of Snail transcription by PARP-1. As expected, the combined doxo-ABT-888 treatment led to an increase in the rate of apoptosis. We show that Snail levels, which were upregulated by doxo treatment, were decreased by ABT-888 addition or by depletion of PARP-1. The involvement of PARP-1 in Snail transcription was further demonstrated by chromatin immunoprecipitation (ChIP) and luciferase assays. The milder upregulation observed upon combined doxo/ABT-888 treatment or PARP-1 depletion appeared to be crucial for releasing PTEN suppression, a key negative regulator of PI3K/Akt activity. The lower activation of PI3K/Akt pathway resulted in the reduction of the survival signaling. Our data reveal potential implications in the use of PARP inhibitors in combination with doxo in therapeutic intervention of some chemoresistant Snail-expressing tumors.

## RESULTS

### ABT-888 treatment and PARP-1 depletion sensitize MDA-MB-231 cells to doxo-induced apoptosis

Doxo has multiple effects on tumor cells, including DNA damage, and prolonged exposure to this agent causes the activation of the apoptotic program in most of cancer cell lines [56]. However, some cells are able to escape apoptosis and develop resistance to drug treatment [57]. We aimed to evaluate the apoptotic response of MDA-MB-231 cells and its dependence on PARP inhibition. Since we obtained similar results using different PARP inhibitors, here we decided to use ABT-888, a novel PARP inhibitor that has been reported to make the tumor more likely to respond to radiation and chemotherapy [58]. MDA-MB-231 cells were treated with either 1 μM doxo, or 0.5 μM ABT-888, or a combination of both drugs for 24–48 h.

**FIGURE 1:**
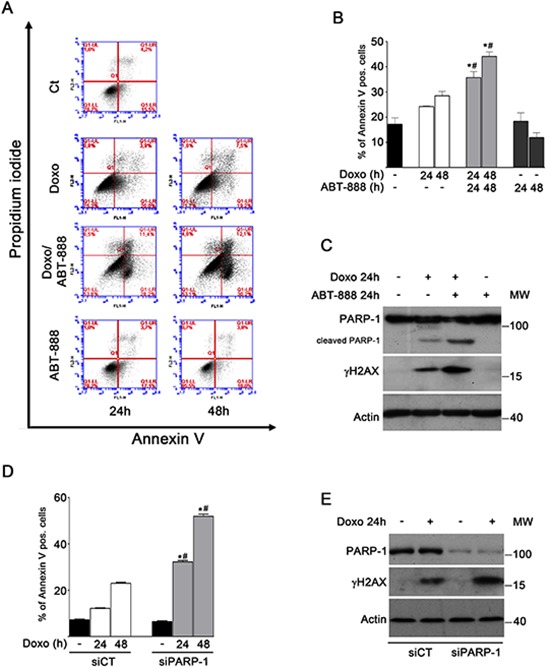
ABT-888 treatment and PARP-1 depletion sensitize MDA-MB-231 cells to doxo-induced apoptosis **A.** Apoptosis was analysed by FACS after treatment of MDA-MB-231 cells with 1 μM doxo and/or 0.5 μM ABT-888 for 24 and 48 h. Panels of a representative experiment are shown. **B.** Annexin V positive cells were counted in the right upper and lower squares. The diagram reports the percentage of Annexin V positive cells in untreated cells (black bar) and after treatment with 1 μM doxo (white bars), 1 μM doxo plus 0.5 μM ABT-888 (light gray bars) or ABT-888 alone (dark gray bars) at the indicated times in relation to total cells. Data represented are the mean+SEM of at least three independent experiments performed in duplicates. Comparisons were made with ANOVA/Turkey's test. *P < 0.05 compared to untreated cells; #*P* < 0.05 compared to cells treated with doxo at 24 h, 48 h respectively. **C.** Levels of cleaved PARP-1 (detected with mAb clone C2-10, Enzo Life Sciences) and γH2AX protein were measured by Western blot analyses in MDA-MB-231 cells treated for 24 h with 1 μM doxo and/or 0.5 μM ABT-888. **D.** Annexin V positive cells were counted in the right upper and lower squares. The diagram reports the percentage of Annexin V positive cells in siCT cells untreated (black bar) or treated with doxo (white bars) and in siPARP-1 cells untreated (black bar) or treated with doxo (light gray bars). Comparisons were made with ANOVA/Turkey's test. *P < 0.05 compared to untreated cell; #*P* < 0.05 compared to cells treated with doxo at 24 h, 48 h respectively. **E.** Levels of PARP-1 and γH2AX protein were measured by Western blot analyses in siCT MDA-MB-231 cells and in siPARP-1 MDA-MB-231cells treated for 24 h with 1 μM doxo.

The presence of apoptotic cells was examined by FACS analysis, counting all the Annexin V positive cells in the right squares of each panel in Figure 1A. A moderate increase in the number of apoptotic cells was evident in doxo-treated *vs* untreated cells at 24 and 48 h Erase this sentence. Conversely, the number of Annexin V positive cells significantly increased at 24 and 48 h of combined treatment with doxo and ABT-888 (up to 2.6-fold *vs* untreated cells) (Figure 1B). Accordingly, when the effect of doxo and ABT-888, alone or in combination, was evaluated in terms of clonogenic ability, the combined treatment resulted in a significant reduction in clonogenic ability of MDA-MB-231 cells (9% survival fraction) with respect to doxo alone (27% survival fraction) or ABT-888 alone (85% survival fraction) (data not shown).

Consistently, only cells exposed to doxo and ABT-888 for 24 h exhibited an increased level of cleaved PARP-1 (detected with clone mAb C2–10), a widely sensitive indicator of caspase-mediated apoptotic cell death, and a concomitant increase in γH2AX formation, which is indicative of an unrepaired damage (Figure 1C).

Then we assessed whether also the depletion of PARP-1 caused the same outcome of the PARP inhibitor ABT-888 in terms of apoptosis. After siRNA-mediated silencing of PARP-1, MDA-MB-231 cells were treated with doxo for 24 and 48 h and apoptosis was evaluated by the Annexin V assay. Graph in Figure 1D shows a significant increase of apoptosis (about 3 fold) in cells silenced for PARP-1 with respect to control cells after doxo treatment. Concomitant with this effect, a higher induction of γH2AX was detectable after 24 h of doxo treatment in siPARP-1 cells with respect to control cells (Figure 1E).

Collectively, these data indicate that reduction of PARP activity may enhance the killing effect of doxo on tumor cells and that this effect may primarily depend on PARP-1.

### PARP-1 activity is required for Snail upregulation in different doxo-treated breast cancer cell lines

**FIGURE 2:**
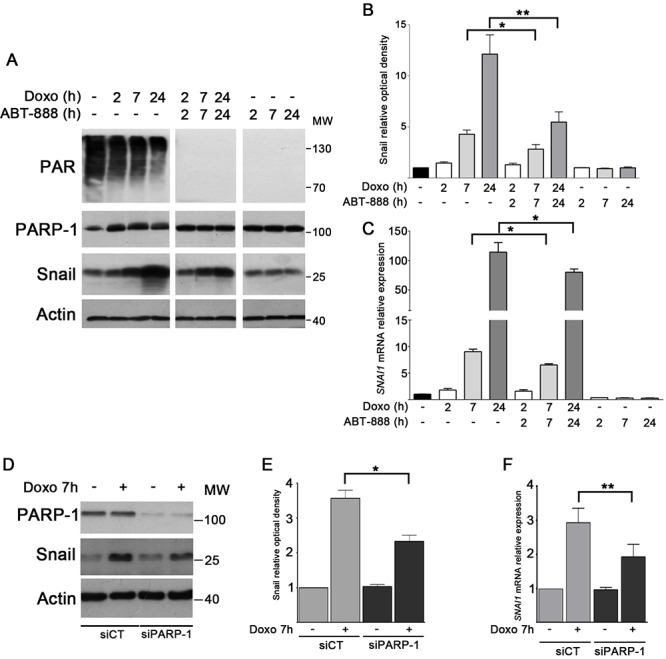
PARP-1 activity is required for Snail upregulation in doxo-treated MDA-MB-231 cells MDA-MB-231 cells were treated with 1 μM doxo, 1 μM doxo plus 0.5 μM ABT-888, 0.5 μM ABT-888 alone at the indicated times. **A.** PAR, PARP-1 (detected with mAb clone F1–23 Enzo Life Sciences) and Snail levels were assessed by Western blot analysis **B.** Graph shows the average densitometry of Snail values normalized to actin, considering Snail level in untreated cells as 1.0. Data represent mean + SEM of three independent experiments. *P < 0.05, **P < 0.01 by Student's *t*-test. **C.** Expression levels of *SNAI1* mRNA were assessed by Real-Time PCR after 2 h (white bars), 7 h (light gray bars) and 24 h (dark gray bars) of treatment and compared to untreated cells (black bar) considered as 1.0. Data represent mean + SEM of at least five independent experiments performed in triplicates. *P < 0.05, **P < 0.01 by Students *t*-test. **D.** Snail protein level was assessed by Western blot in MDA-MB-231 cells silenced for PARP-1 after treatment with 1 μM doxo for 7 h. **E.** Graph shows the average densitometry of Snail values normalized to actin, considering Snail level in untreated cells as 1.0. Data represent mean + SEM of three independent experiments. *P < 0.05 by Student's *t*-test. **F.** Levels of *SNAI1mRNA* in MDA-MB-231 cells silenced for PARP-1 (dark gray bars) after treatment with 1 μM doxo for 7 h in relation with cells transfected with siCT (light gray bars). Data represent mean + SEM of three independent experiments performed in triplicates. **P < 0.01 by Student's *t*-test.

Although the mechanisms of apoptosis are complex, there is accumulating evidence to suggest that Snail is an important component in defining the response of tumor cells to chemotherapeutic agents [15]. Since the PARylation process has been correlated to the modulation of Snail level [24, 27], we aimed to evaluate its role in Snail expression during doxo treatment in MDA-MB-231. Cells were treated with either doxo 1 μM, or ABT-888 0.5 μM or a combination of both drugs at different times (2 h, 7 h and 24 h). As shown by Western blot analyses (Figure 2A), Snail levels increased in a time-dependent manner upon treatment with doxo, while the combined treatment with both doxo and ABT-888 resulted in a milder induction. This decrease in Snail level is significant, as revealed by densitometric analyses in Figure 2B. PAR level is quite high in our untreated cells probably because of the higher rate of basal PARP-1 activity, often observed in cancer cells [59]. However, the efficacy of PARP inhibition was confirmed by PAR disappearance in presence of ABT-888 alone or in combination with doxo. Real-Time PCR analyses were performed to determine whether the variation of Snail protein was correlated to changes of its mRNA expression. Figure 2C shows that *SNAI1* mRNA levels significantly upregulated in a time-dependent manner after doxo treatment, reaching very high levels at 24 h (114 fold increase *vs* untreated cells) while cotreated cells showed a decrease in *SNAI1* mRNA levels (30 + 7% at 24 h). The *SNAI1* mRNA levels of ABT-888 treated cells did not show a significant difference compared to untreated cells.

To assess whether PARP-1 was involved in the modulation of Snail induction after doxo treatment, MDA-MB-231 cells were silenced for PARP-1 by 48 h of siRNA transfection and then treated with doxo for 7 h. Western blot analyses (Figure 2D) indicate a considerable reduction of Snail level after doxo treatment in PARP-1-depleted cells with respect to control cells. Densitometric analysis shows that Snail decrease is significant (Figure 2E). Again, Real-Time PCR analysis indicates that the sharp increase of *SNAI1* mRNA level upon doxo treatment in siCT cells was significantly reduced in PARP-1-silenced cells (Figure 2F).

Then we compared the effects of doxo and ABT-888 treatments on Snail expression in the two additional TNBC cell lines MDA-MB-468 and MDA-MB-157, and in the epithelial cell line MCF-7. Regarding the triple negative cells, while MDA-MB-468 cells exhibited a time-dependent consistent increase in Snail protein and mRNA level in response to doxo (Supplementary Figure 1A and 1B), MDA-MB-157 cells exhibited a significant (5 fold) increase only at 24 h of doxo treatment with respect to untreated cells (data not shown). Notably, both triple-negative cancer cells showed a significant reduction of the doxo-induced Snail level in cells cotreated with ABT-888 and doxo, suggesting a positive effect of the PARP inhibitor as antitumor agent in this subset of breast tumors. In spite of the low basal Snail level, also MCF-7 cell line evidenced an increase in Snail protein and mRNA level upon doxo treatment, (less consistent at mRNA level than in MDA-MB-231 cells), which was counteracted by ABT-888 cotreatment (Supplementary Figure 1C, 1D).

Collectively, these results indicate that PARP-1 regulates Snail expression following doxo treatment.

### PARP activity affects PARP-1 binding and histone H3 modifications profiles at *SNAI1* locus in doxo-exposed MDA-MB-231 cells

**FIGURE 3:**
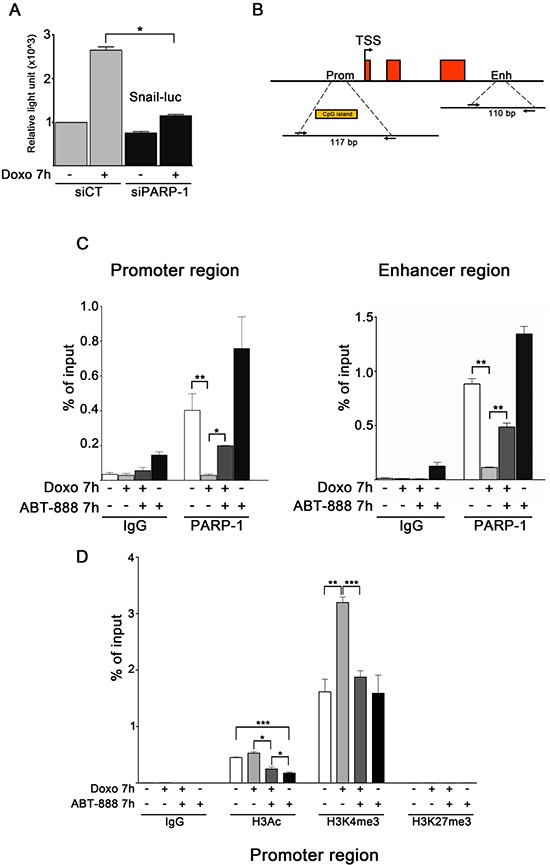
PARP activity affects PARP-1 binding and histone H3 modifications profiles at *SNAI1* locus in doxo-exposed MDA-MB-231 cells **A.** Dual-luciferase assay of MDA-MB-231 cells transfected with a Snail reporter plasmid and siRNA to knockdown PARP-1. Results are presented as variation of light units measured in siCT cells (light gray bars) exposed to doxo and in untreated and doxo-exposed siPARP-1 cells (black bars) relatively to the activity of siCT untreated cells, set as 1.0. Data (mean and SEM) are representative of at least three independent experiments. *P < 0.05 by Student's *t*-test. **B.** Schematic view of the human *SNAI1* gene, comprised of three exons. Among the regulatory elements, a CpG island in the promoter region and an enhancer, corresponding to a DNase hypersensitive site, are highly conserved. The position of the primers pair used to amplify the indicated sequence of the promoter region (length 117bp) and enhancer region (length 110bp) are reported. **C.** MDA-MB-231 cells, treated for 7 hours with 1 μM doxo, 0.5 μM ABT-888, 1 μM doxo plus 0.5 μM ABT-888, were fixed and lysed. ChIP assays for PARP-1 were conducted and DNA isolated from PARP-1 IPs was used in Real-Time PCR to amplify the indicated genomic loci on promoter (left panel) and on enhancer (right panel). DNA coprecipitated with control IgG was also amplified to control aspecific signal. **D.** MDA-MB-231 cells, treated for 7 hours with 1 μM doxo, 0.5 μM ABT-888, 1 μM doxo plus 0.5 μM ABT-888, were fixed and lysed ChIP assays for H3Ac, H3K4me3 and H3K27me3 were conducted. DNA was isolated from IPs and used in Real-Time PCR to amplify the indicated genomic locus on *SNAI1* promoter. DNA coprecipitated with control IgG was also amplified to control aspecific signal. Each experiment of Real-Time PCR was conducted in triplicates and depicted as the average of immunoprecipitated signal to input signal and SEM. Data represent averages of three experiments.*P < 0.05, **P < 0.01, ***P < 0.001 by Students *t*-test.

Snail promoter contains an E-box motif, defined as the binding site of PARP-1 located within the Snail-ILK responsive element (SIRE) sequence [27, 60]. To investigate whether Snail promoter activity directly responds to PARP-1, the transfection with a luciferase reporter construct [61] carrying the human Snail promoter was combined with the silencing of PARP-1 in MDA-MB-231 cells (Figure 3A). The depletion of PARP-1 expression at 48 h from siRNA transfection was controlled by Western blot (data not shown). Snail promoter activity was measured after exposure of transfected cells to doxo for 7 h. We noticed that doxo treatment resulted in an increase in Snail promoter activity in control samples, which was not observed in PARP-1 depleted samples. This result shows that PARP-1 depletion negatively regulated Snail promoter activation induced by doxo and this is in agreement with the expression of endogenous Snail, which was downregulated at protein and mRNA level (Figure 2D, 2F).

To obtain information about the direct role played by PARP-1 in *SNAI1* expression in MDA-MB-231 cells, ChIP assays were performed to analyse PARP-1 interactions with the promoter and the 3′ enhancer (Figure 3B), a region that is reported to control the expression of Snail in melanoma cells A375 [62]. Analyses were performed in MDA-MB-231 cells treated for 7 h with either doxo 1 μM, or ABT-888 0.5 μM or a combination of both drugs. As shown in Figure 3C, PARP-1 occupied the promoter and enhancer regions both in presence and in absence of ABT-888. This binding was abrogated upon doxo treatment. Doxo/ABT-888 cotreatment counteracted PARP-1 removal from the *SNAI1* promoter/enhancer regions. To confirm the specificity of our ChIP assays, we carried out a positive control consisting in the amplification of a region on *ITPR1* promoter, positively regulated by PARP-1 (Supplementary Figure 2) [63]. Thus, PARP-1 is not present on the promoter/enhancer regions when Snail is upregulated upon doxo treatment while it reappears on those regions when Snail induction is lowered by the doxo/ABT-888 cotreatment.

ChIP assays were performed in order to characterize the histone modification profile at the *SNAI1* promoter and its response to treatments. Figure 3D shows that H3K27 trimethylation (H3K27me3), found in facultatively repressed genes, was not present in any of the analyzed conditions. Conversely, acetylated H3 (H3Ac) and H3K4 trimethylation (H3K4me3), which are associated to actively transcribed promoters, occupied the promoter indicating its “on” state. H3K4me3 increased significantly as the gene was induced by doxo treatment with respect to untreated, cotreated or ABT-888-treated cells.

Collectively, these results reveal an important role of PARP activity in defining the right chromatin context of *SNAI1* promoter in response to doxo and suggest a direct involvement of PARP-1.

### PARP-1 inhibition/depletion antagonizes doxo-induced downregulation of *PTEN* in MDA-MB-231 cells and decreases Akt activity

**FIGURE 4:**
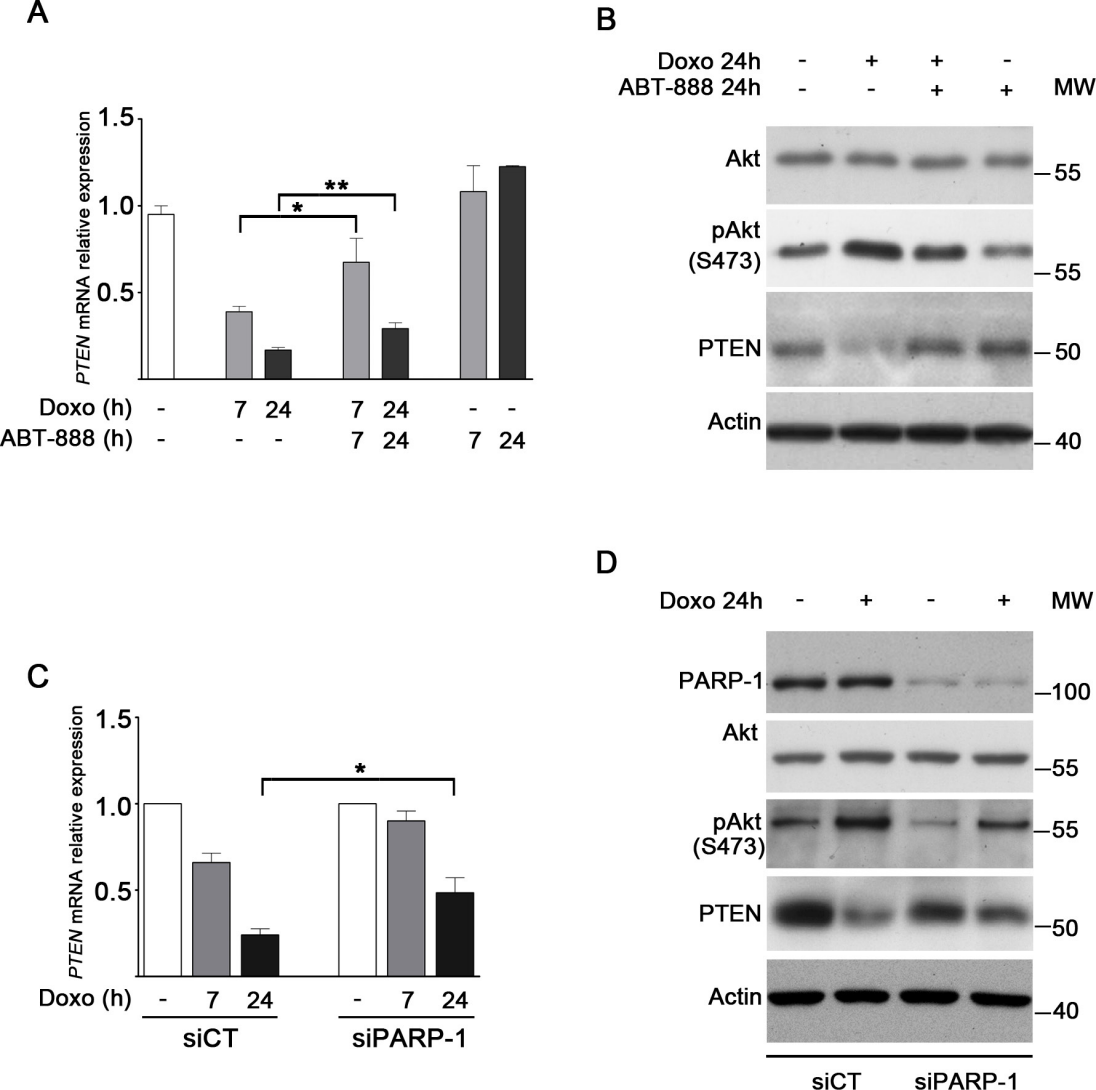
PARP-1 inhibition/depletion antagonizes doxo-induced downregulation of PTEN in MDA-MB-231 cells and decreases Akt activity **A.** Expression levels of *PTEN* after treatment with 1 μM doxo, 1 μM doxo plus 0.5 μM ABT-888 or ABT-888 for 7 h (light gray bars) or 24 h (dark gray bars) in relation to untreated cells (white bar), considered as 1.0. Data represent mean+SEM of at least five independent experiments performed in triplicates. *P < 0.05, **P < 0.01 by Student's *t*-test. **B.** Levels of Akt, phospho-Akt, PTEN and γH2AX protein were measured by Western blot analyses in MDA-MB-231 cells treated for 24 h with 1 μM doxo and/or 0.5 μM ABT-888 as indicated. **C.** Expression levels of *PTEN* after treatment of siCT and siPARP-1 MDA-MB-231 cells with 1 μM doxo for 7 h (light gray bars) or 24 h (dark gray bars) in relation to untreated siCT and siPARP-1 cells (white bars), considered as 1.0. Data represent mean+SEM of three independent experiments performed in triplicates. *P < 0.05 by Student's *t*-test. **D.** Levels of PARP-1, Akt, phospho-Akt, and PTEN protein were measured by Western blot analyses in siCT and siPARP-1 MDA-MB-231 cells treated for 24 h with 1 μM doxo.

Snail represses pro-apoptotic genes in the DNA damage response pathway, promoting cell survival [14, 20]. Previous results showed that, upon DNA damage, Snail physically associates with the *PTEN* promoter and inhibits its expression [21]. We asked whether the doxo-induced upregulation of Snail was able to repress *PTEN* expression in MDA-MB-231 cells and whether ABT-888 treatment or PARP-1 depletion, by antagonizing Snail upregulation, could play a role in modulating *PTEN* expression. Real-Time PCR analysis (Figure 4A) showed a drastic decrease in *PTEN* expression in cells exposed to doxo that was significantly counteracted by cotreatment with ABT-888. The *PTEN* mRNA levels following doxo and doxo/ABT-888 treatment resulted inversely correlated to the Snail level which is in agreement with the reported function of Snail on *PTEN* repression.

PTEN is a phosphatase, which provokes Akt dephosporylation and its inhibition [64]. Therefore PTEN suppression should cause Akt activation. Western blot analysis evidenced (Figure 4B) that the treatment of MDA-MB-231 cells with doxo increased Akt(Ser473) phosphorylation, in accordance with the reduction of PTEN which was confirmed at protein level too. Conversely, the cotreatment with doxo/ABT-888, which allowed the recovery of PTEN at mRNA and protein level, induced a decrease in Akt activation thus favouring apoptosis.

In line with the result of the combined treatment, we showed that the depletion of PARP-1 caused a lower suppressive effect on *PTEN* expression with respect to control cells upon doxo treatment (Figure 4C). Consistently, Western blot analysis shows that the increased Akt(Ser473) phosphorylation detected in control cells upon doxo treatment was reduced in PARP-1-depleted cells (Figure 4D).

### Snail knockdown sensitizes MDA-MB-231 cells to doxo-induced apoptosis and allows recovery of *PTEN* expression

**FIGURE 5:**
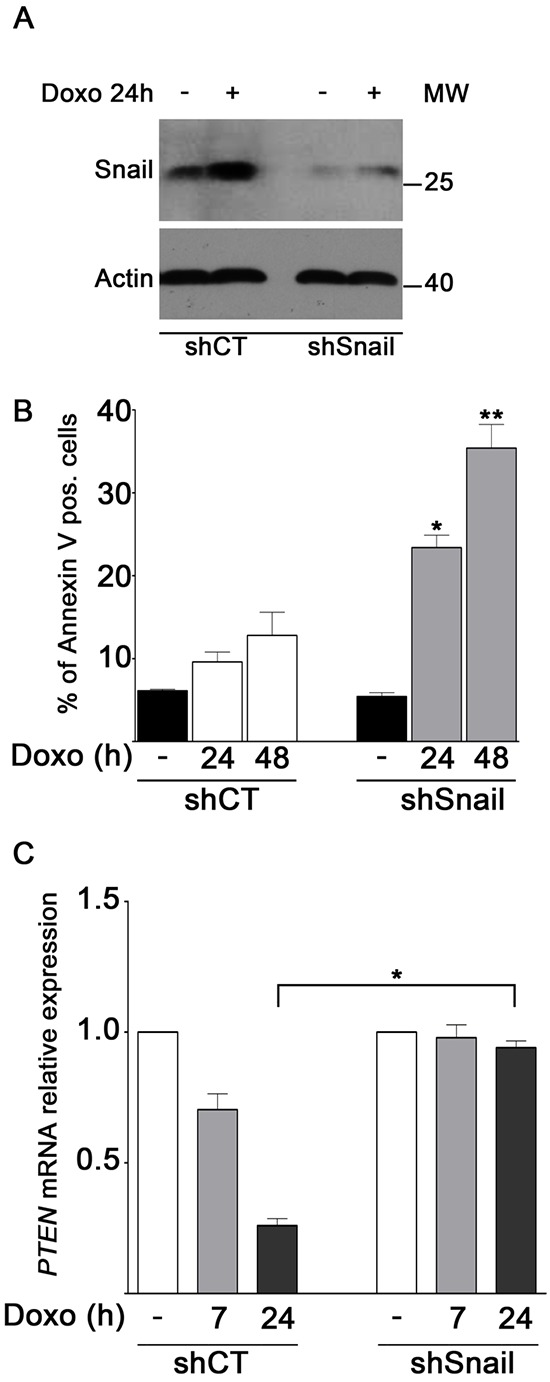
Snail knockdown sensitizes MDA-MB-231 cells to doxo-induced apoptosis and allows recovery of PTEN expression **A.** Levels of Snail protein were measured by Western blot analyses in shCT and shSnail MDA-MB-231 cells treated for 24 h with 1 μM doxo **B.** Annexin V positive cells were counted in the right upper and lower squares. The diagram reports the percentage of Annexin V positive cells in untreated shCT and shSnail cells (black bars) and after treatment of shCT (white bars) and shSnail cells (light gray bars) with 1 μM doxo at the indicated times in relation to total cells. Data represented are the mean+SEM of three independent experiments performed in duplicates. Comparisons were made with ANOVA/Turkey's test. *P < 0.05, **P < 0.01 compared to cells treated with doxo at 24 h, 48 h respectively. **C.** Expression levels of *PTEN* after treatment of shCT and shSnail MDA-MB-231 cells with 1 μM doxo for 7 h (light gray bars) or 24 h (dark gray bars) in relation to untreated shCT and shSnail cells (white bars), considered as 1.0. Data represent mean+SEM of three independent experiments performed in triplicates. *P < 0.05 by Student's *t*-test.

To confirm that the rate of apoptosis in MDA-MB-231 cells depended on Snail levels, MDA-MB-231 cells were transfected with shRNA-Snail or shRNA-CT plasmids. Figure 5A shows that depletion of endogenous Snail was efficient as Snail signal resulted low after doxo treatment too. A significant increase in the number of Annexin V positive cells after treatment with doxo at 24–48 h was observed in Snail-depleted cells with respect to control cells (around 3 fold) (Figure 5B). These data demonstrate that Snail deficiency is required for efficient apoptosis in doxo-treated cells and support the idea that PARP-1 inhibition/depletion sensitizes MDA-MB-231 cells by limiting Snail upregulation. Interestingly, Real-Time PCR analysis (Figure 5C) showed that, upon doxo treatment, the clear *PTEN* downregulation of control cells was not detectable in Snail-depleted cells. This result is in agreement with the presence of high Snail levels and their repressive function on PTEN expression in control cells exposed to doxo.

## DISCUSSION

Recent studies have evidenced that PARP inhibitors may chemosensitize TNBC cell lines [53–55]. Also known is that Snail can regulate cancer cell survival [14, 15] and that PARP-1 may be involved in its regulation [24, 27].

Here we aim to investigate the possible cross-talk between PARP-1 and Snail in MDA-MB-231 cells, a model of Snail-expressing TNBC cell line, in response to the chemotherapeutic agent doxorubicin.

First, we showed that the PARP inhibitor ABT-888 or PARP-1 depletion increases the sensitivity to doxo. Consistently, the combined doxo/ABT-888 treatment caused a concomitant increase in γH2AX formation associated to a significant reduction in clonogenic ability. Then we investigated whether this chemosensitizing effect was linked to the control of Snail expression exerted by PARP-1. Our results demonstrate that both ABT-888 treatment and PARP-1 depletion were able to counteract the strong upregulation of Snail observed upon treatment with doxo. Thus, PARP-1 seems to play a positive role in the regulation of Snail expression, thereby favoring the survival of cancer cells. One attractive hypothesis is that activation of PARP-1 may grant the low cytotoxicity of MDA-MB-231 cells by promoting not only the DNA repair process but also Snail induction. Conversely, the decrease of PARP-1 activity may allow efficient apoptosis by blocking DNA repair and reducing Snail level. Interestingly, Snail is still upregulated upon combined treatment, indicating that Snail expression did not depend only on PARP-1. Other pathways may control Snail level in DNA damage response [65, 66] or route it in different processes from cell survival such as the EMT [26].

Results on the different cell lines led to the important conclusion that PARP activity always provides a contribution in Snail expression, albeit variable in dependence of the cell line [59]. In fact, we observed a trend of Snail level to rise after doxo and to fall after doxo/ABT-888 treatment both in the TNBC cell lines and in MCF-7 cells. Our preliminary results (data not shown) and recent literature [67, 68] indicate that Snail functions are not necessarily related to cell survival in p53-proficient MCF-7 cells. In fact, in MCF-7 cells Snail is implicated in regulating various targets of the EMT process unlike the p53-mutated TNBC cell lines where Snail acts as an anti-apoptotic factor [15, 17]. Thus in MCF-7 cells, PARP-1 might participate to the EMT process through Snail modulation.

Further, the silencing of PARP-1 in MDA-MB-231 cells has demonstrated that PARP-1 was crucial for the induction of the *SNAI1* promoter transcriptional activity. More information was obtained by ChIP assays, which investigated the binding of PARP-1 to specific regions at the *SNAI1* locus. In untreated or ABT-888-treated cells, PARP-1 occupied the *SNAI1* promoter/enhancer regions and its presence was correlated to Snail basal transcription. This suggests that PARP-1, independently of its activity [69], may act as gene-specific coregulator providing features of permissive/active chromatin. On the contrary, PARP-1 no longer occupied the *SNAI1* promoter and enhancer regions upon doxo treatment, indicating that PARP-1 activation and its displacement from DNA are important events in the induction mechanism of Snail expression. Consistently, PARP inhibition reestablished PARP-1 occupancy of the DNA loci after combined doxo/ABT-888 treatment, giving rise to a lower Snail upregulation.

ChIP analyses for chromatin marks showed that H3K27me3 was not present on *SNAI1* promoter in any of the analyzed conditions while active histone modifications (H3Ac and H3K4me3) were well represented, indicating the “on” state of the promoter. We speculate that PARP-1 allows the recruitment of positive modifiers such as histone acetylases and histone methyltransferases in absence of any damaging stimulus. The importance of the basal activity of PARP-1 in H3 acetylation is supported by H3Ac depletion arisen from exposure to ABT-888 either in presence or absence of doxo. As the gene was induced by doxo treatment, H3K4me3 increased significantly, suggesting the possibility that newly synthesized PARs on PARP-1 may block the access of some negative effector to the promoter, such as the lysine demethylase 5B (KDM5B) which is therefore kept far from the promoter [63].

Further experiments were carried out to individuate pro-apoptotic targets of Snail affected by PARP inhibition/depletion. We focused our attention on different targets from p53, since in MDA-MB-231 cells, p53 results to be highly expressed and its function impaired. According to recent papers [21, 70], Snail can activate Akt by repressing the PI3K negative regulator PTEN, a direct Snail target. Our Real-Time PCR results show that in cells exposed to doxo the strong *PTEN* downregulation was counteracted by cotreatment with ABT-888 or by depletion of PARP-1. This result suggests the intervention of Snail on *PTEN* expression, since high levels of Snail elicited by doxo cause *PTEN* downregulation whereas lower Snail levels are in accordance with a lower suppressive effect on *PTEN* expression. As expected, *PTEN* suppression was associated to an increase of Akt phosphorylation in doxo treated cells. Conversely, the milder *PTEN* downregulation was correlated to a reduction of Akt phosphorylation in doxo treated cells either exposed to ABT-888 or depleted of PARP-1. Therefore, we suggest that PARP-1, by regulating at transcriptional level the expression of the oncoprotein Snail, controls Akt phosphorylation through targeting PTEN expression.

Since we have shown that Snail contributes to activate survival pathways, its reduction, by offsetting these pathways, should result in higher cytotoxicity to the doxo treatment. Effectively, Snail deficiency of MDA-MB-231 cells showed an increased sensitivity to doxo-induced apoptosis. Moreover, Snail deficiency opposed to *PTEN* suppression elicited by doxo treatment, supporting the inhibitory role of Snail on PTEN expression. This result recalls the enhanced apoptosis obtained following PARP inhibition/depletion and suggests that the downregulation of Snail level may represent the crucial event to reach apoptosis.

**FIGURE 6:**
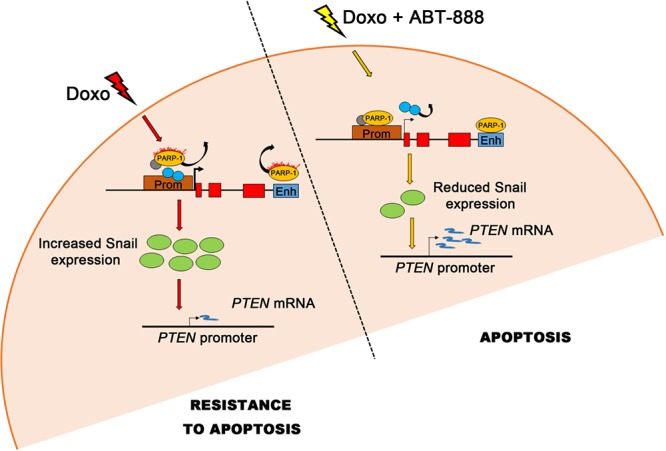
Model of Snail transcriptional regulation by PARP-1 and effect of the differential Snail expression on apoptosis in MDA-MB-231 cells The presence or absence of PARP-1 on the *SNAI1* promoter/enhancer, which depends on the rate of PARP activity, affects the activity of *SNAI1* promoter (see Discussion). After the doxorubicin treatment (left side), PARP activity increases and PARP-1 detaches from DNA probably bringing away repressive factors (gray circle) from the promoter while other positive transcription factors (blue circles) may bind it. This event causes a strong increase in Snail transcription (green circles) and the consequent repression of its target gene *PTEN* which results in resistance to apoptosis. After the treatment with doxo/ABT-888 (right side), PARP activity decreases and PARP-1 can bind the *SNAI1* promoter/enhancer possibly causing the release of positive transcription factors from the promoter (light blue circles). The Snail transcription is lower, leading to a less efficacious repression of Snail on *PTEN* transcription and to a significant recovery of the apoptotic process.

In conclusion, PARP-1 works as a kind of switch modulating Snail expression and hence the response to doxo treatment, as depicted in the model shown in Figure 6. The low apoptotic rate observed in MDA-MB-231 cells exposed to doxo is correlated to the high expression levels of Snail oncoprotein. PARP-1 activation, which coincides with its displacement from *SNAI1* locus, is required for Snail induction. The increased apoptotic rate is related to a lower upregulation of Snail. PARP-1 seats on the promoter and controls, through its basal activity, Snail transcription.

Moreover, we showed that the lower upregulation of Snail is a fundamental event to the occurrence of apoptosis because it causes the recovery of PTEN expression. Further studies are required to clarify this molecular mechanism in detail.

In summary, the data of this study indicate that the lowering of Snail levels in cancer cells may represent a new crucial mechanism by which PARP inhibitors act sensitizing to chemotherapeutic drugs. In considering the mechanism of action of PARP inhibitors, it is important to keep in mind not only their function on DNA damage [71], but also on the regulation of cancer-related genes expression. We believe that more knowledge on the effect of PARP inhibitors in specific cell lines will allow to improve the outcome of specific subgroups of breast cancer patients.

## MATERIALS AND METHODS

### Cell cultures and treatments

Breast cancer cell lines MDA-MB-231, MDA-MB-468, MDA-MB-157, MCF-7 (ATCC) were maintained in high glucose DMEM (Gibco, BRL) supplemented with 10% FBS (Gibco, BRL). All culture solutions were supplemented with 2 mM L-glutamine (Sigma-Aldrich) and 50 units/ml Penicillin-Streptomycin (Sigma-Aldrich). Treatments of cells were performed preparing mediums containing doxorubicin (Sigma-Aldrich) at a final concentration of 1 μM and ABT-888 (Veliparib) (Enzo Life Sciences) at a final concentration of 0.5 μM.

### Colony forming assay

To evaluate cell colony-forming ability, exponentially growing MDA-MB-231 cells were treated with doxo 0.5 μM or ABT-888 0.5 μM alone or in combination for 2 hours. At the end of treatment, aliquots of cell suspension from each sample were seeded into 60-mm Petri dishes with complete medium and incubated for three weeks. Colonies were stained with 2% methylene blue in 95% ethanol and counted. Percentage of colonies arising from surviving treated cells was calculated relative to colonies arising from untreated control cells.

### Analysis of apoptosis by Annexin V staining

Induction of apoptosis was assessed by measuring Annexin V binding to externalized phosphatidylserine, as previously described [72]. Briefly, cells were washed twice with PBS and resuspended in binding buffer 10 mM Hepes/NaOH pH 7.4, 140 mM NaCl, 2.5 mM CaCl_2_ (Sigma Chemical Co.). FITC conjugated Annexin V (Roche Diagnostic Corp.) was added at a final concentration of 1 μg/ml. The mixture was incubated at room temperature for 15 min in the dark. Stained cells were analyzed by flow cytometry using a Accuri C6 flow cytometer (Becton Dickinson), while simultaneously assessing membrane integrity by propidium iodide (PI) (0.25 μg/ml) exclusion. Samples were analyzed using the CFlow Plus software.

### Western blot analysis

Total cell lysates were prepared in RIPA buffer (50 mM Tris-HCl at pH 7.4, 150 mM NaCl, 1% NP-40, 0.5% sodium deoxycholate, 0.1% SDS and 1 mM EDTA) added with sodium orthovanadate 3 mM, NaF 100 mM supplemented with protease inhibitors (Sigma-Aldrich). Lysates were kept on ice for 25 min and then centrifuged at 16000 g for 30 min at 4°C. Supernatants were collected and quantified by Bradford protein assay reagent (Biorad). 25 μg of proteins were loaded and separated by SDS-PAGE and electroblotted onto nitrocellulose membrane (Hybond^TM^ ECL^TM^ GE Healthcare). Immunoblots probed with the specific antibodies were developed using ECL or ECL Plus chemiluminescence reaction. The following antibodies were used: Snail (L70G2) (Cell Signaling), PARP-1 (clone C2–10 able to detect the 89kDa cleaved fragment of PARP-1; Enzo Life Sciences), PARP-1 (clone F1–23; Enzo Life Sciences), PAR (clone 10HA; Trevigen), Actin (Sigma-Aldrich), Akt (Cell Signaling), Phospho-Akt (Ser473) (Cell Signaling), PTEN (Cell Signaling), Phospho-Histone H2AX (Ser139) (Millipore).

### Quantitative real-time PCR

RNA was extracted with the RNeasy micro kit (Qiagen), and treated with RNase-free DNase (Qiagen). The RNA concentration and purity (260/280 and 260/230 ratios) was analyzed using a ND-1000 Spectrophotometer (NanoDrop Technologies). Total RNA was subjected to retrotranscription using SuperScript VILO cDNA Synthesis Kit (Life Technologies, Invitrogen). Quantitative Real-Time PCR reactions were performed with KAPA^TM^ SYBR^®^ FAST qPCR Kits (KAPA BIOSYSTEMS). The sense oligonucleotide for *SNAI1* was 5′-CTCTAATCCAGAGTTTACCTTC-3′ and the antisense was 5′-GACAGAGTCCCAGATGAG-3′. The sense oligonucleotide for *PTEN* was 5′-ATGACAGCCATCATCAAAGAG-3′ and the antisense was 5′-GTGCCACTGGTCTATAATCCA-3′. Gene expression analysis was performed using the comparative cycle threshold method with *GAPDH* for normalization. The sense oligonucleotide for *GAPDH* was 5′-CCAGTGACGTTCCCGTTCAGC-3′ and the antisense was 5′-CCCATCACCATCTCCCAGGAG-3′.

### Transfection of MDA-MB-231 cells

MDA-MB-231 were transfected with Lipofectamine 2000 reagent (Life Technologies, Invitrogen) adopting the manufacturer's protocol. For silencing experiments, cells were transfected with siGENOME SMARTpool *PARP-1* and siGENOME Non-Targeting siRNA (Thermo Scientific, Dharmacon) at a final concentration of 50 nM. To generate Snail-deficient cells, MDA-MB-231 were transfected with shRNA containing specific oligonucleotide sequences against EGFP (22 nt) or human Snail (19 nt: 5′-GATGCACATCCGAAGCCAC-3′) cloned into the pSuperior-Puro vector (Oligoengine). The selection was obtained with puromycin 1 μg/ml (Invitrogen) for 2–4 weeks. Appropriate expression levels of Snail were confirmed by immunoblotting.

### Chromatin immunoprecipitation (ChIP)

ChIP assays were performed as previously described [73]. The antibodies used for immunoprecipitation were: normal rabbit IgG (Santa Cruz), polyclonal antibody for PARP-1 (Enzo Lifescience), H3Ac (Millipore), H3K4me3 (Millipore) and H3K27me3 (Millipore). The sense oligonucleotide used to amplify the promoter region was 5′-AACCCCGCCTCGGAGGAGT-3′ and the antisense oligonucleotide was 5′-CCAATCGGAGGCTCGTCT-3′. Real-Time PCR assays were carried out to amplify the enhancer region as already described [62]. The sense oligonucleotide used was FW 5′-GAGCAGCCCTTAATGACTTG-3′ and the antisense oligonucleotide was 5′-CCCAACTCCCTAACTTCCC-3′. The sense oligonucleotide used to amplify the promoter region of *ITPR1* was 5′-ACTGAGGTCGCGGTTTGTAT-3′ and the antisense oligonucleotide was 5′-AAGGAGCCGTGTTGTGACTT-3′ [63].

### Luciferase assay

For the luciferase assays, cells were transfected with Lipofectamine 2000 (Life Technologies, Invitrogen) according to the manufacturer's instructions. Cells were seeded in 12-well culture plates at a concentration of 2 × 10^5^ cells/well and incubated for 24 h prior to cotransfection involving luciferase reporter constructs. The vector containing luciferase under the control of human Snail promoter corresponding to sequences −598 to −578 (sense) and +92 to +72 (antisense) was obtained as indicated [61].

### Statistical analysis

Statistical tests were performed using GraphPad Prism 6.0 and the number of replicates (n) performed are reported in figure legends. Data were considered to be statistically significant if *P < 0.05.

## SUPPLEMENTARY FIGURES


