# Supplementary material for: PARP inhibitor ABT-888 affects response of MDA-MB-231 cells to doxorubicin treatment, targeting Snail expression
Source: Oncotarget. 2015 Apr 13;6(17):15008–21. doi: 10.18632/oncotarget.3634 (PMC4558132; doi:10.18632/oncotarget.3634)
Supplement: Supplementary file 1 [file oncotarget-06-15008-s001.pdf]

SUPPLEMENTARY FIGURES

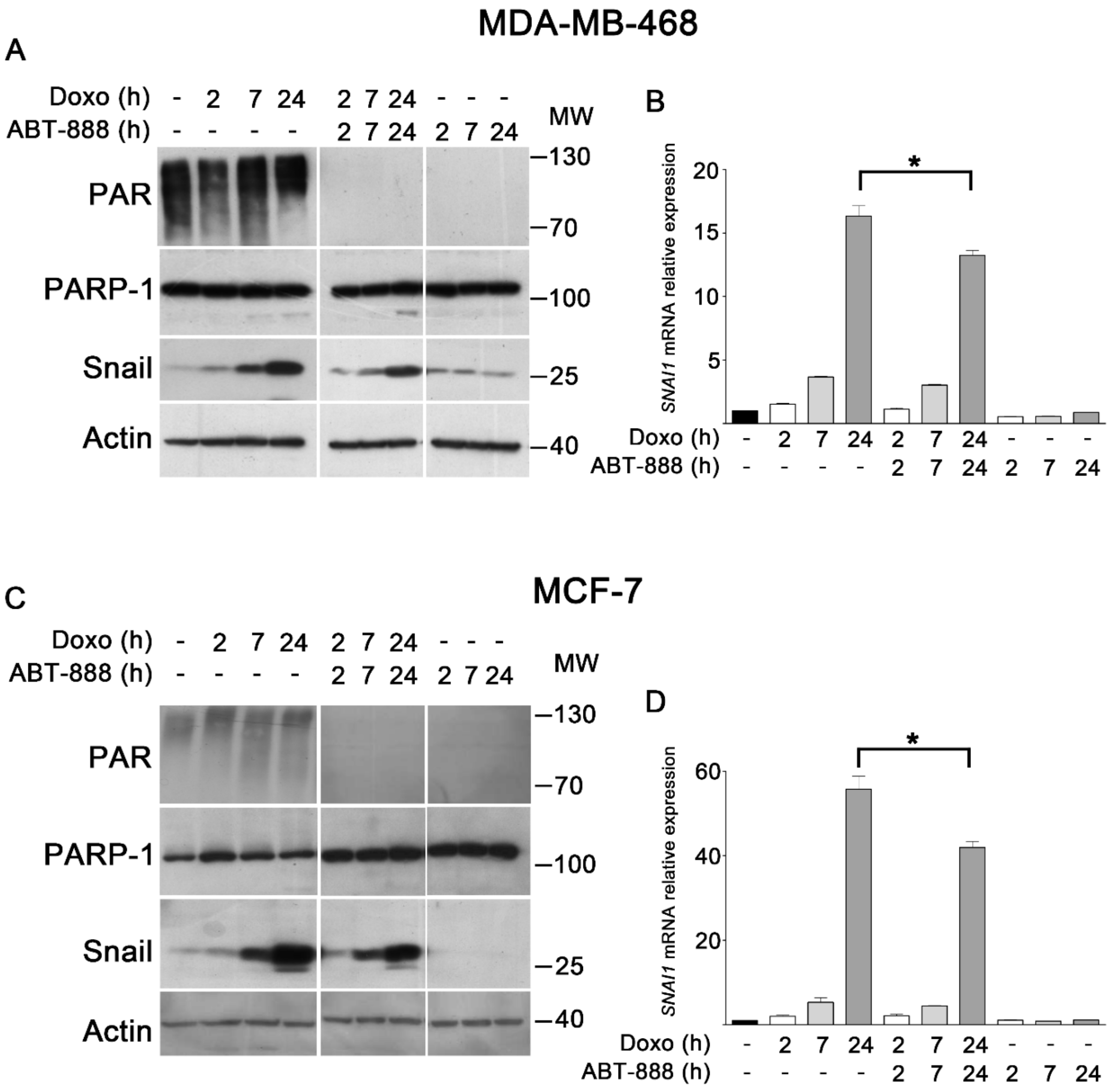

Supplementary Figure S1: PARP-1 activity is required for Snail upregulation in doxo-treated MDA-MB-468 and MCF-7 breast cancer cell lines. MDA-MB-468 and MCF-7 cells were treated with 1 µM doxo, 1 µM doxo *plus* 0.5 µM ABT-888, 0.5 µM ABT-888 alone at the indicated times. **A, C.** PAR, PARP-1 (detected with mAb C2-10; Enzo Life Sciences) and Snail levels were assessed by Western blot analysis **B, D.** Expression levels of *SNAI1* mRNA were assessed by Real-Time PCR after 2h (*white bars*), 7h (*light gray bars*) and 24h (*dark gray bars*) of treatment and compared to untreated cells (*black bar*) considered as 1.0. Data represent mean + SEM of three independent experiments performed in triplicates. *$P < 0.05$ by Students *t*-test.

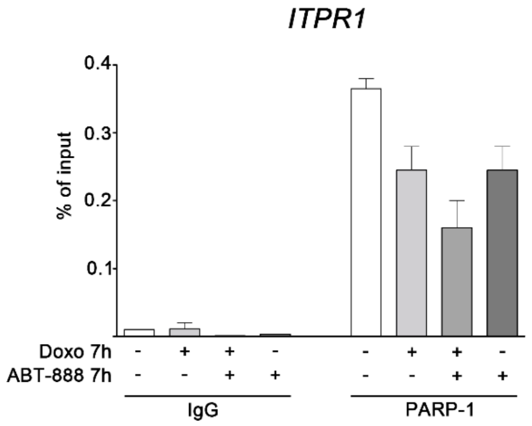

Supplementary Figure S2: ChIP analyses of PARP-1 on the *ITPR1* promoter. MDA-MB-231 cells, treated for 7 hours with 1 µM doxo, 0.5 µM ABT-888, 1 µM doxo *plus* 0.5 µM ABT-888, were fixed and lysed. ChIP assays for PARP-1 were conducted and DNA isolated from PARP-1 IPs was used in Real-Time PCR to amplify a 100bp region on *ITPR1* promoter. DNA coprecipitated with control IgG was also amplified to control aspecific signal.
